# Supplementary material for: TRS: a method for determining transcript termini from RNAtag-seq sequencing data
Source: Nat Commun. 2023 Nov 29;14:7843. doi: 10.1038/s41467-023-43534-2 (PMC10687069; doi:10.1038/s41467-023-43534-2)
Supplement: Supplementary file 1 — Supplementary Information File [file 41467_2023_43534_MOESM1_ESM.pdf]

Supplemental Information

TRS: a method for determining transcript termini from RNAtag-seq sequencing data

Amir Bar, Liron Argaman, Michal Eldar and Hanah Margalit

Department of Microbiology and Molecular Genetics
IMRIC, Faculty of Medicine, The Hebrew University of Jerusalem,
Jerusalem, 9112102, ISRAEL

TABLE OF CONTENTS

Supplementary Text

Supplementary Tables

Supplementary Table 1. Number of reads in sequencing libraries of the current study

Supplementary Table 2. Oligonucleotides used in this study

Supplementary Figures

Supplementary Fig. 1. Simulated gene read pattern in RNAtag-seq data

Supplementary Fig. 2. Overlap between experimentally determined 3' termini and 3' termini obtained by applying TRS to published RNAtag-seq data

Supplementary Fig. 3. Assessment of 3' termini detected by TRS applied to RNAtag-seq data

Supplementary Fig. 4. Sequence and structure analysis of unique 3' termini and of read starts

Supplementary Fig. 5. Relative frequency of putative falsely detected 3' termini due to PCR duplicates by their genomic annotation

Supplementary Fig. 6. Overlap between primary 3' termini determined by TRS applied to previously published RNAtag-seq of *E. coli* by other research groups and known 3' termini

Supplementary Fig. 7. Repertoire of 3' termini identified by TRS in other bacteria

Supplementary Fig. 8. 3' UTR-derived transcripts identified in the *E. coli* K-12 LB RNAtag-seq dataset

Supplementary Fig. 9. Feasibility of using paired-end read starts to simultaneously determine transcript 5' and 3' boundaries

Supplementary Fig. 10. The relationship between the mean and the variance of the number of read starts

Supplementary Fig. 11. The effect of the read coverage on the value of the statistic $R_{i,j}$

Supplementary Fig. 12. The cumulative distribution function of the mean statistic $\overline{R_i}$

Supplementary Fig. 13. Effect of sequencing depth on the identified 3' termini

Supplementary Fig. 14. Effect of window size on identified 3' termini

Supplementary Fig. 15. Effect of downstream distance on identified 3' termini

Supplementary Fig. 16. Distribution of distances between dominant and prominent positions of statistically significant $\overline{R_\iota}$ peaks

Supplementary References

Supplementary Text

Parameter estimation for $L_{i,j}$ distribution

To estimate the parameters for $L_{i,j}$ distribution, we used the number of read starts in the downstream d positions (which are also used to evaluate $D_{i,j}$). We assume that the number of read starts in each of the 'd' positions are identically and independently distributed. Thus, the distribution of the expected number of read starts per position is the sample mean, $m = \frac{D_{i,j}}{d}$. Similarly, we estimated the variance of the number of read starts in the downstream window (s^2). We observed that there is a direct relation between the log of the mean and the log of the variance across different positions in the genome (Supplementary Fig. 10). Therefore, a more accurate assessment of the variance can be achieved by using a regression model to estimate the variance directly from the mean. In practice we used a linear regression with a second order polynomial kernel to estimate the variance from the mean, that is $\log s^2 = \beta_1 + \beta_2 \cdot \log m + \beta_3 \cdot (\log m)^2$.

Effect of sequencing depth on identified 3' termini

To quantify the effect of the sequencing depth on the identified 3' termini, we sampled each of the three replicates at different sampling probabilities (ranging from 0.2 to 0.9) and repeated this process 10 times. Thus, for each sampling probability we have 10 different samples of the three biological replicates. By using this setup, we were able to measure the variability in determined 3' termini among the read sets sampled with a certain probability, as well as the variability in determined 3' termini in read sets sampled with different probabilities. As expected, the number of 3' termini identified, and their overlap decrease as the sampling probability decreases (Supplementary Fig. 13a-b). To explore whether this process can introduce artificial 3' termini, we checked for each 3' terminus in each sample whether it was found in the original dataset (no sampling), and the number of samples it was found in (Supplementary Fig. 13c-d). We found that most 3' termini were found in multiple samples, and that 3' termini that varied among the samples were identified in the original data. Only several 3' termini (between 2 to 24 on

average, depending on sample probability) were not identified in the original data and potentially represent artificial 3' termini.

Effect of parameter choice on identified 3' termini

To understand how identified termini are affected by the choice of parameters, we ran the algorithm on *E. coli* K-12 LB RNAtag-seq data for different values of the local window width (W) and the distance downstream the tested position (D), while fixing the rest of the parameters. This analysis included termini that passed the statistical tests in all three libraries. We studied the effect of local window width for values of 0 to 7 and found an increase in the number of identified 3' termini with increasing values of W, until reaching a plateau at W=3 (Supplementary Fig. 14a). For window width ≥3, the sets of determined 3' termini are highly consistent (Supplementary Fig. 14b). Similarly, we ran the algorithm for 11 equally spaced values of D between 20 nucleotides (nt) and 70 nt (corresponding to 17 nt and 67 nt, respectively, which are these values minus the selected window size 'W') and measured the number of determined 3' termini and the overlap between them (Supplementary Fig. 15a-c). We found that the number of 3' termini decreases as we increase D and stabilizes at about a distance of 60 nt. Overall, most 3' termini were identified with all D values (Supplementary Fig. 15b) and for every pair of tested values the identified 3' termini were highly similar, with minimal overlap coefficient of 0.95 and minimal Jaccard index of 0.76 (Supplementary Fig. 15c). There were no differences in the numbers of determined mature and premature 3' termini for the various values of D (Supplementary Fig. 15d).

Testing for statistical significance of peaks within each library

The peaks identified by our pipeline are computed for the mean statistic $\overline{R_\iota}$. This has two implications: first, the highest position of the peak, hereinafter the dominant position, is not necessarily the position with the highest number of read starts, since the statistic measures local "readthrough". In fact, it appears that in many cases the dominant position is located a few nucleotides downstream the position with the highest number of read starts (Supplementary Fig. 16). Secondly, the dominant position might not be the best position to use in the statistical test, when there are inconsistencies among libraries as to

the exact position with the highest number of read starts. Therefore, when we test for statistical significance of a peak in each library, we apply it to the position with the highest number of read starts within the peak in the library.

TRS is applicable to term-seq data

To verify that TRS can identify 3' termini also in term-seq data, we applied it to the term-seq libraries of Dar and Sorek[1], resulting in 1465 3' termini (Supplementary Data 1-2). Then, these positions were compared to the 1088 3' termini reported in the original study. We identified 952 overlapping 3' termini (~88%) between the two datasets, a statistically significant overlap (p-value≤1E-2070 by hypergeometric test). It is of note that in the original study, Dar and Sorek focused only on primary 3' termini. Hence, they analyzed the term-seq data heuristically, relying on the genomic annotation of *E. coli* and requiring a minimal number of four read starts on average across three replicates to determine a 3' terminus downstream a stop codon of a gene. This has two implications: First, 3' termini other than primary are inherently ignored by their pipeline but not by TRS, and thus all the detected non-primary termini are expected to be unique to the data of term-seq analyzed by TRS. Secondly, focusing on specific genomic regions (downstream stop codons of genes) allowed the authors to set a low threshold of four read starts, which is not applicable when considering the entire genome. Thus, it is possible that 3' termini that were not detected by TRS are those supported by a low number of read starts. To test this conjecture, we checked for the reason 3' termini unique to Dar and Sorek were not re-discovered by TRS: whether they had low read coverage or whether they did not pass the statistical test. Strikingly, 119 out of the 136 undetected 3' termini had low coverage, below the minimal read start count threshold we set. Overall, we conclude that TRS can be reliably applied to term-seq data.

RNAtag-seq and term-seq experiments for bacteria grown on minimal medium

We grew cultures of three biological replicates of *E. coli* K-12 MG1655 on minimal medium (EG), extracted RNA, and divided each RNA sample to two aliquots, to one we applied RNAtag-seq and to the other term-seq. We applied TRS to the read data and determined 3' termini, as described in the main text. We obtained 1810 3' termini from the

RNAtag-seq data and 1885 3' termini from the term-seq data. By comparing 3' termini in the two datasets, we found a statistically significant overlap of 1261 3' termini (p-value≤1E-2512 by hypergeometric test; Supplementary Fig. 3a). In addition, we found 549 3' termini unique to the RNAtag-seq dataset and 624 3' termini unique to the term-seq dataset.

Precision

The precision of TRS applied to RNAtag-seq data is defined as the number of true positive 3' termini (TP) divided by the total number of 3' termini identified in the RNAtag-seq data, where true positive 3' termini are determined by TRS applied to the term-seq data. We found that 3' termini identified in the RNAtag-seq data had a precision of 1261 / 1809 (70%). Furthermore, if we consider additional 3' termini identified in term-seq data of cells grown in rich medium and 3' termini reported by previous studies[1-3] (Supplementary Fig. 3b), we find that 370 out of the 549 unique 3' termini are also supported, and the precision is 90%. When focusing only on primary 3' termini (Fig. 3 in the main paper), the precision rises to 98%.

Sensitivity

The sensitivity of TRS applied to RNAtag-seq is defined as the number of TP out of the total number of 3' termini identified in the term-seq data, which is 1261 / 1885 (67%). When focusing only primary 3' termini the sensitivity is 83%.

All in all, these results are consistent with the results reported in the main text for cells grown in rich medium. We conclude that TRS applied to RNAtag-seq identifies 3' termini in high precision and sensitivity compared to TRS applied to term-seq data.

<u>TRS applied to *E. coli* RNAtag-seq data from other studies correctly identifies primary 3' termini</u>

To verify that TRS can be applied to RNAtag-seq data generated by other labs as well, we downloaded previously published RNAtag-seq data from four different studies[2, 4-6]. These data were generated for *E. coli* K-12 MG1655[2, 4, 6], or a related strain for which

the read data was mapped to the same reference genome[5], grown to either exponential phase or to early stationary phase (Supplementary Data 1). We applied TRS to determine 3' termini in each dataset and each 3' terminus was assigned a genomic annotation (Supplementary Data 2).

Since TRS showed best performance in determination of primary 3' termini, for the purpose of the analysis, we compared the overlap of primary 3' termini between each of the datasets and primary 3' termini previously published by Dar and Sorek[1] and Ju *et al.*[3]. We report a statistically significant overlap between the 3' termini determined for each of the RNAtag-seq datasets and the previously published 3' termini ($p \leq 1E-568$ in all these analyses; Supplementary Fig. 6). The statistically significant high overlap rates, despite differences in strains and growth conditions, strongly support that TRS has high efficacy in determining primary 3' termini in RNAtag-seq data.

<u>3' termini determined by TRS applied to RNAtag-seq and term-seq datasets of the same RNA samples are consistent</u>

In addition to our study in *E. coli*, described in the main text, there was another study that applied RNAtag-seq and term-seq to the same RNA samples of *E. coli*[2] (Supplementary Data 1). We applied TRS to these two datasets and determined 3' termini in each of them (Supplementary Data 2). We then carried out comparative analysis, revealing high consistency in the 3' termini determined by TRS applied to the RNAtag-seq and to the term-seq datasets. 84% of all the 3' termini identified in the term-seq data were identified in the RNAtag-seq data, and ~96% of primary 3' termini identified in the term-seq data were identified in the RNAtag-seq data. Of note, this study included only two replicates for each of the RNAtag-seq and term-seq experiments, which is not optimal for TRS and often may lead to a lower number of identified 3' termini (1335 and 742 in RNAtag-seq and term-seq datasets, respectively). In addition, this study is somewhat unique in capturing relatively lower abundance of primary 3' termini (381 and 252 in the RNAtag-seq and term-seq and data, respectively) compared to internal 3' termini. This finding is consistent with the results of the original study.

Identification of 3’ termini in other bacteria by applying TRS to available RNAtag-seq data

While it is evident that TRS applied to RNAtag-seq data can be used to determine genuine 3’ termini in *E. coli*, it would be highly informative to examine its performance on data of other bacteria. To verify that TRS can identify 3’ termini in RNAtag-seq data of other bacteria, we applied it to previously published RNAtag-seq data of four different bacteria: *K. pneumoniae, S. enterica, S. flexneri,* and *L. monocytogenes*[7] (Supplementary Data 1). For L. *monocytogenes* EGD-e, a corresponding term-seq data was also available to us from a previous study of this bacterium grown in similar conditions[8] (Supplementary Data 1). We applied TRS to both datasets and identified 1178 and 1092 3’ termini in the RNAtag-seq and the term-seq datasets, respectively (Supplementary Data 2). Consistent with comparisons of 3’ termini identified in RNAtag-seq and term-seq data in *E. coli* (Table 1 in main text), 70% of all term-seq 3’ termini were also identified by RNAtag-seq and 88% of the primary 3’ termini (Supplementary Fig. 7a-b). As shown in Supplementary Fig. 7c, most of the 3’ termini were annotated as primary 3’ termini. We examined the distribution of 3’ termini classes identified in the other three bacteria and found that these were in accord with the distributions observed in *E. coli* data (Supplementary Fig. 7d). Indeed, most of the 3’ termini were annotated as primary 3’ termini and the relative frequencies of the terminus classes were similar to those computed for *E. coli* K-12, suggesting that TRS can be also applied to other bacteria as well. These results suggest that TRS applied to RNAtag-seq is not limited to specific bacteria, and RNAtag-seq data can be utilized for the determination of 3’ termini in any bacterium.

Identification of orthologous genes between bacteria

For each bacterium, the nucleotide sequence of each protein coding gene was extracted according to the reference genome and the annotation file (see Supplementary Data 1). These sequences were used as input to EggNOG v5.0 server (emapper-2.1.19)[9], a database of orthology relationships.

Comparison to known cleavage sites

To estimate how many 3' termini are associated with cleavage events, we compared our set of LB RNAtag-seq 3' termini with cleavage site maps of RNase E[10] and RNase III in *E. coli*[11]. We considered a 3' terminus overlapping a cleavage site if they were located at most 10 nucleotides apart. In total, out of 1003 RNase III and 6997 RNase E cleavage sites, only 56 and 108 cleavage sites, respectively, overlapped 3' termini. This may suggest that cleavage sites mostly result in unstable fragments or that they are further processed, and thus are unlikely to be detected by methods that capture 3' termini.

<u>Upper bound estimation of PCR duplicates</u>

One of the pitfalls in analysis of RNA-seq data is the occurrence of PCR duplicates that falsely increase the number of reads starting at a position. While the TRS algorithm considers 3' termini identified in multiple replicates, lowering the risk of false detections, it would be informative to assess the possible effect of PCR duplicates. While in single-end data, as analyzed in this study, there is no practical way to estimate PCR duplicates, they can be removed from paired-end sequencing data[12, 13]. Hence, we used paired-end RNAtag-seq data for this analysis. To estimate an upper bound to the number of 3' termini possibly generated by PCR duplicates, we applied TRS analysis to sequencing libraries with and without computationally removing PCR duplicates (samtools1.17 rmdup[14]). 3' termini identified without the removal of PCR duplicates, but not after their removal, are considered as 3' termini that may have been falsely detected due to PCR duplicates. We applied this analysis to two paired-end sequencing datasets of *E. coli*, one of libraries that were amplified by five cycles of PCR[4] and one of libraries that were amplified by nine cycles of PCR[15]. Without the removal of PCR duplicates, application of TRS to the sequencing libraries of the two studies has resulted in 2108 3' termini in Melamed et al. data[4] and 1716 3' termini in Bar et al. data[15]. Repeating the analysis following the removal of PCR duplicates resulted in exclusion of 234 and 255 3' termini, respectively. Some of the excluded 3' termini due to alluded PCR duplications overlapped previously documented 3' termini[1-3], and therefore were excluded from this analysis. Following the exclusion of known 3' termini, our analysis identified 169 3' termini in Melamed *et al.* data[4] and 160 3' termini in Bar *et al.*[15], which could be due to PCR duplications (comprising

8.02% and 9.32% of total 3' termini), respectively. These overall fractions are even lower for primary 3' termini (~2% in the two datasets).

Overall, we conclude that more caution should be taken when considering a premature termination or a cleavage site when analyzing single-end data, in which PCR duplicates cannot be removed. Yet, since it is still possible that the removed fragments are genuinely biological copies, we consider these estimates as an upper bound.

Structure prediction upstream to 3' termini

To predict the RNA secondary structure upstream a 3' terminus, the nucleotide sequence of the 51 positions upstream to the 3' terminus and the position itself (total of 52 positions) were extracted. The secondary structure was predicted using RNAfold 2.4.14[16] with the default parameters, and the free energy of the structure was recorded.

Supplementary Tables

Supplementary Table 1. Number of reads in sequencing libraries of the current study

Library name*	Total # of reads	# of reads after processing	# of reads after processing / Total reads	# of mapped reads (filtered from BAM)	# of mapped reads / # of reads after processing
**LB1_RNAtag-seq**	24841238	20067356	0.8078	18123746	0.9031
**LB2_RNAtag-seq**	38653691	29569372	0.765	26527611	0.8971
**LB3_RNAtag-seq**	29900635	23621776	0.79	21314660	0.9023
**LB1_Term-seq**	22553371	14731496	0.6532	13231947	0.8982
**LB2_Term-seq**	23970387	17148826	0.7154	15720312	0.9167
**LB3_Term-seq**	15866879	11125088	0.7012	10143509	0.9118
**EG1_RNAtag-seq**	38838863	29869517	0.7691	26933932	0.9017
**EG2_RNAtag-seq**	31686107	25138313	0.7934	22701526	0.9031
**EG3_RNAtag-seq**	27709285	21963853	0.7927	19813609	0.9021
**EG1_Term-seq**	24662944	18986531	0.7698	17819660	0.9385
**EG2_Term-seq**	28635885	20788568	0.726	19346723	0.9306
**EG3_Term-seq**	26812368	20006263	0.7462	18715063	0.9355

*LB: rich medium. EG: minimal medium.

Supplementary Table 2. Oligonucleotides used in this study

Name	Sequence*
3' barcoded adapter	/5Phos/ATCCCGCGGAGATCGGAAGAGCGTCGTGTA/3ddC/
3' barcoded adapter	/5Phos/AGTCTGGCGAGATCGGAAGAGCGTCGTGTA/3ddC/
3' barcoded adapter	/5Phos/AGGTCCTCTAGATCGGAAGAGCGTCGTGTA/3ddC/
3' barcoded adapter	/5Phos/ACGGCACTTAGATCGGAAGAGCGTCGTGTA/3ddC/
3' barcoded adapter	/5Phos/ATACAACATAGATCGGAAGAGCGTCGTGTA/3ddC/
3' barcoded adapter	/5Phos/ATCATCGTGAGATCGGAAGAGCGTCGTGTA/3ddC/
3' barcoded adapter	/5Phos/AACATTATTAGATCGGAAGAGCGTCGTGTA/3ddC/
3' barcoded adapter	/5Phos/AAAGTGTTGAGATCGGAAGAGCGTCGTGTA/3ddC/
3' barcoded adapter	/5Phos/AAGAATTATAGATCGGAAGAGCGTCGTGTA/3ddC/
3' barcoded adapter	/5Phos/AATATGGACAGATCGGAAGAGCGTCGTGTA/3ddC/
3' barcoded adapter	/5Phos/AATCACTTGAGATCGGAAGAGCGTCGTGTA/3ddC/
3' barcoded adapter	/5Phos/ACCAAGTCGAGATCGGAAGAGCGTCGTGTA/3ddC/
Reverse Transcription primer	TACACGACGCTCTTCCGAT
3' adapter for second ligation	/5Phos/AGATCGGAAGAGCACACGTCTG/3ddC/
P5 primer	AATGATACGGCGACCACCGAGATCTACACTCTTTCCCTACACGACGCTCTTCCGATCT
P7 indexed primer A001	CAAGCAGAAGACGGCATACGAGATCGTGATGTGACTGGAGTTCAGACGTGTGCTCTTCCGATC
P7 indexed primer A002	CAAGCAGAAGACGGCATACGAGATACATCGGTGACTGGAGTTCAGACGTGTGCTCTTCCGATC
rpsA probe	CCAGCAGCTGATCGCGCTC
rpsL probe	GGCCATCTGGCTGGAGTGC
uspA probe 1	GCTAGCGTTTCTTGTGCCGTTACGTCA
uspA probe 2	GTGTTTATAAGCCATAGTGTTACTCCTTCCATAAA

*/5Phos/ denotes 5' phosphorylation. /3ddC/ denotes 3' dideoxy cytosine.

Supplementary Figures

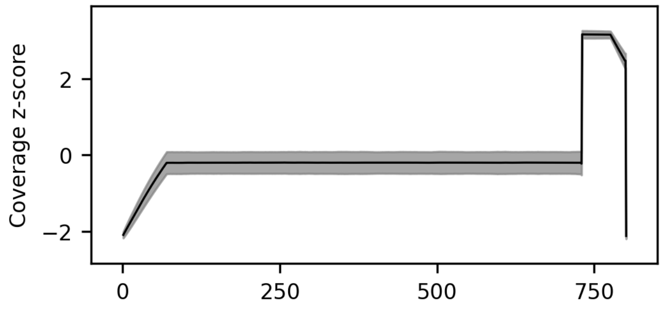

Supplementary Fig. 1. Simulated gene read pattern in RNAtag-seq data. 10,000 read coverage simulations of an 800 nucleotide-long gene were performed. Briefly, in each iteration, the transcript copy number was sampled from a uniform distribution between 50 to 1,000. Next, each copy was fragmented at random positions and corresponding sequencing reads were generated (see Methods in main text). Finally, the number of sequencing reads covering each position was measured. Since the transcript copy number varies between iterations, in each iteration we normalize the coverage by computing the z-score per position (mean and standard deviation are computed over the coverage along the gene). Shown are the mean normalized (z-score) coverage (black line) surrounded by two standard deviations (gray region) of the 10,000 iterations.

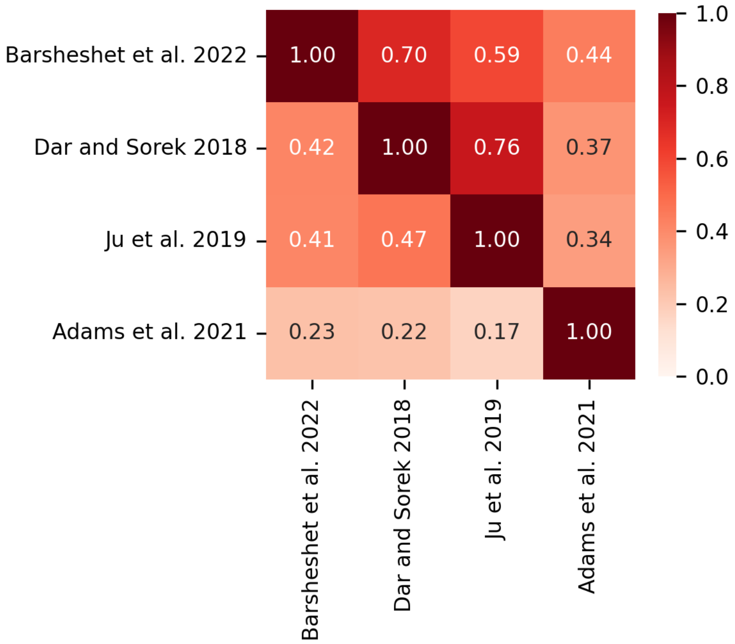

Supplementary Fig. 2. Overlap between experimentally determined 3' termini and 3' termini obtained by applying TRS to published RNAtag-seq data[1]. Heatmap showing the overlap between every two datasets of 3' termini by two measures: The Jaccard index (lower triangle), which considers the intersection of datasets compared to their union, and the overlap coefficient (upper triangle), which considers the intersection of datasets compared to the smaller dataset size.

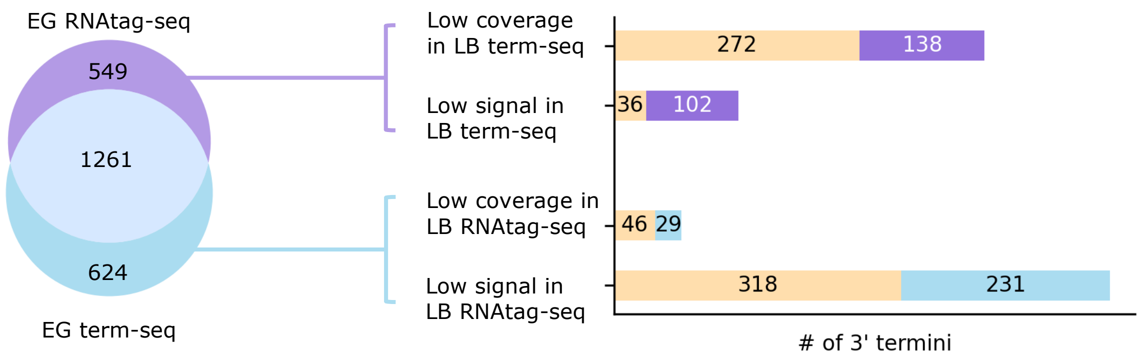

Supplementary Fig. 3. Assessment of 3’ termini detected by TRS applied to RNAtag-seq data (related to Fig. 5a in the main text). Comparison between the 3’ termini identified by TRS applied to data of RNAtag-seq (purple) and term-seq (light blue) conducted on the same RNA samples from cells grown in minimal (EG) medium. 3’ termini unique to each protocol were further analyzed for the possible reason they were not detected by the other protocol, either due to low coverage or due to statistically insignificant p-value (low signal). The distributions of 3’ termini into these two categories are presented as colored bars, where the number of 3’ termini supported by previous studies[1-3] or in our LB term-seq libraries are marked in yellow.

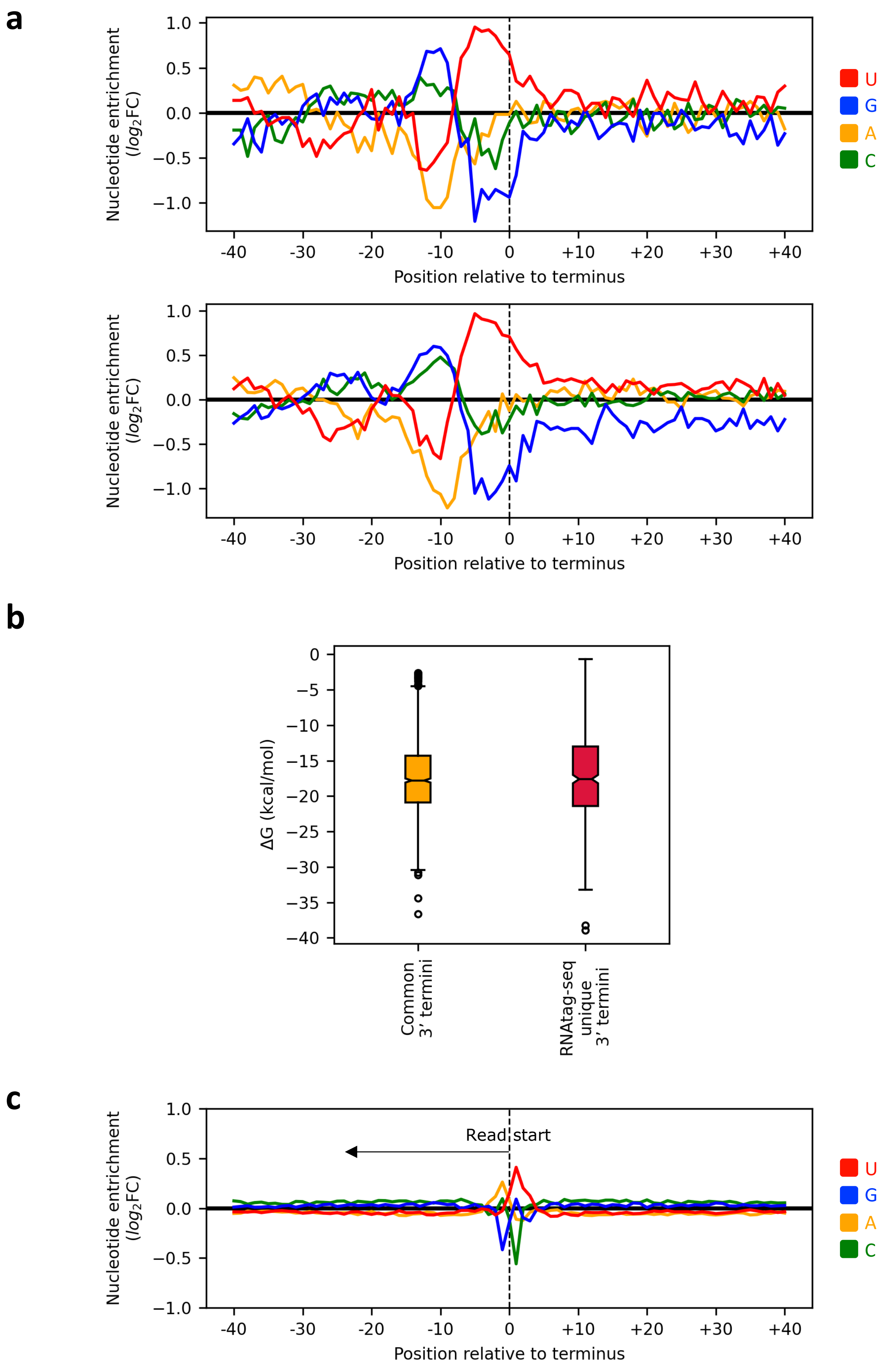
a
Nucleotide entrichment
(log_2FC)
Position relative to terminus
U
G
A
C
b
ΔG (kcal/mol)
Common
3' termini
RNAtag-seq
unique
3' termini
c
Read start

Supplementary Fig. 4. Sequence and structure analysis of unique 3' termini and of mapped read starts. 3' termini that were identified in the LB RNAtag-seq dataset but not in the LB term-seq dataset (RNAtag-seq unique 3' termini) were extracted, as well as 3' termini that were identified in both datasets (common 3' termini). These groups of 3' termini were analyzed for two features: (a) The sequences flanking the reported 3' termini were extracted and aligned at the 3' terminus position. Presented is the nucleotide enrichment relative to the 3' terminus in RNAtag-seq unique 3' termini (upper panel) and the common 3' termini (lower panel). Nucleotide enrichment was computed as the $\log_2$ fold change between the observed nucleotide frequency and background nucleotide frequency, which is 0.25 in *E. coli*. (b) The sequences upstream the 3' termini were extracted, and their structures were computationally determined (Supplementary Text). Presented is the free energy distribution of the structures upstream to the RNAtag-seq unique 3' termini and upstream to the common 3' termini. (c) The sequences flanking the mapped read start positions within CDSs were extracted and aligned at the read start position. Nucleotide enrichment was computed as in (a).

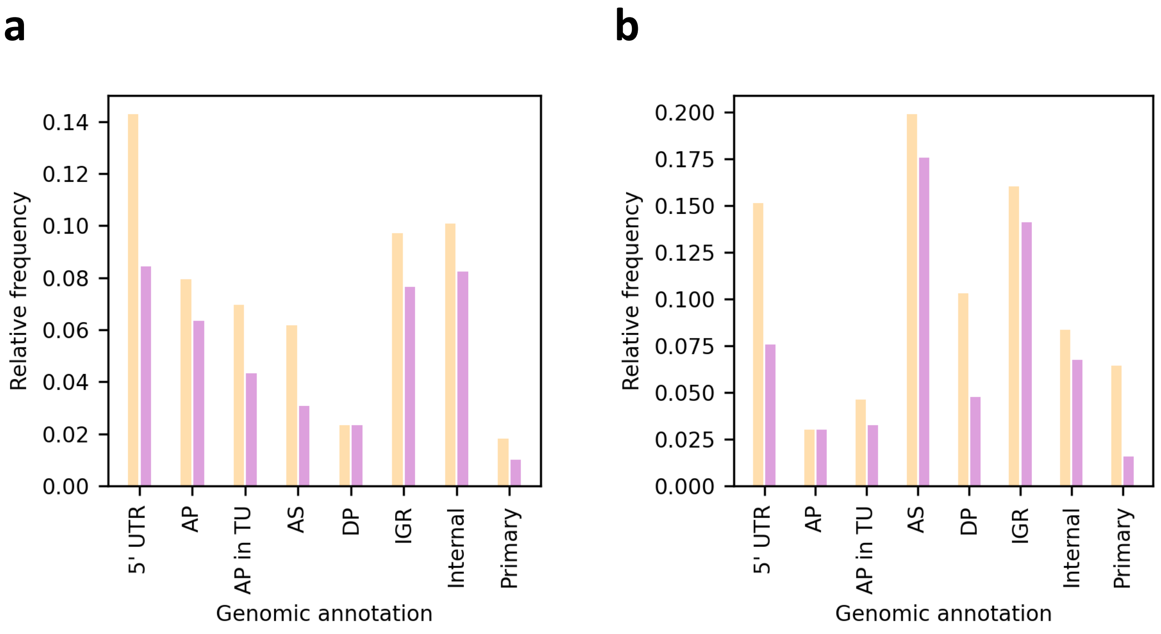

Supplementary Fig. 5. Relative frequency of putative falsely detected 3' termini due to PCR duplicates by their genomic annotation. The number of 3' termini assigned to each genomic annotation, which might have been falsely detected due to PCR duplicates was recorded. Two sets of putatively falsely detected 3' termini were analyzed: 1) all determined 3' termini. 2) a sub-group including only 3' termini that were not reported in previous publications[1-3]. Presented in bars are the relative frequencies of these 3' termini in each genomic annotation for two datasets: (a) Melamed *et al.* 2020[4] and (b) Bar *et al.* 2021[15]. The relative frequencies before (yellow) and after (purple) the exclusion of previously reported 3' termini are presented by orange and purple bars, respectively. The 3' termini annotation and abbreviations are according to Fig. 3 in the main text.

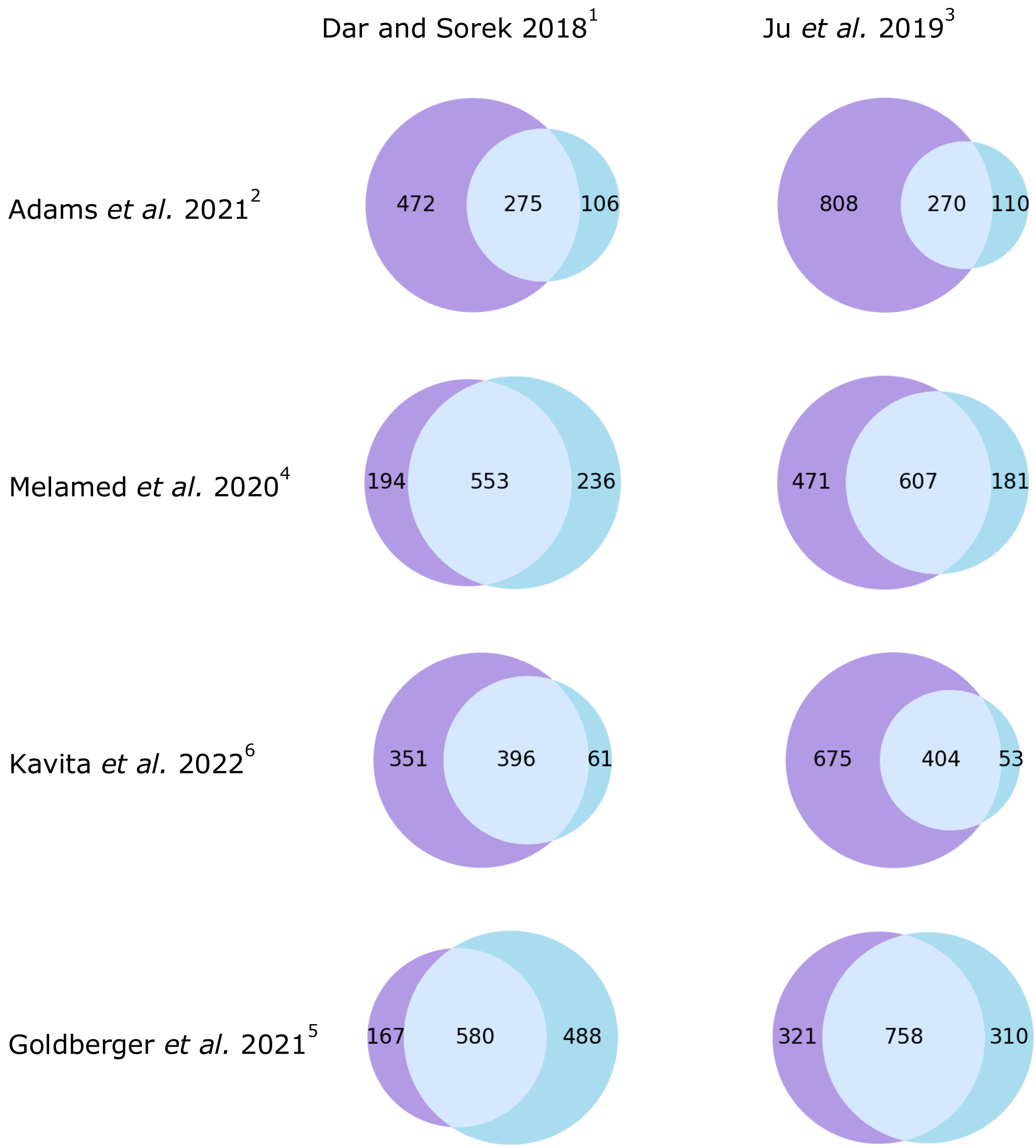

Supplementary Fig. 6. Overlap between primary 3' termini determined by TRS applied to previously published RNAtag-seq of *E. coli* from other research groups and known 3' termini. Primary 3' termini determined by TRS applied to previously published RNAtag-seq data from other labs[2, 4-6] were compared to primary 3' termini reported by experimental approaches[1, 3]. Each row presents the overlap of each of the previously published RNAtag-seq datasets with each of the known 3' termini datasets (columns). Unique 3' termini among the known 3' terminus group are shown in purple,

overlapping 3’ termini in light blue, and unique 3’ termini determined in the RNAtag-seq datasets in blue.

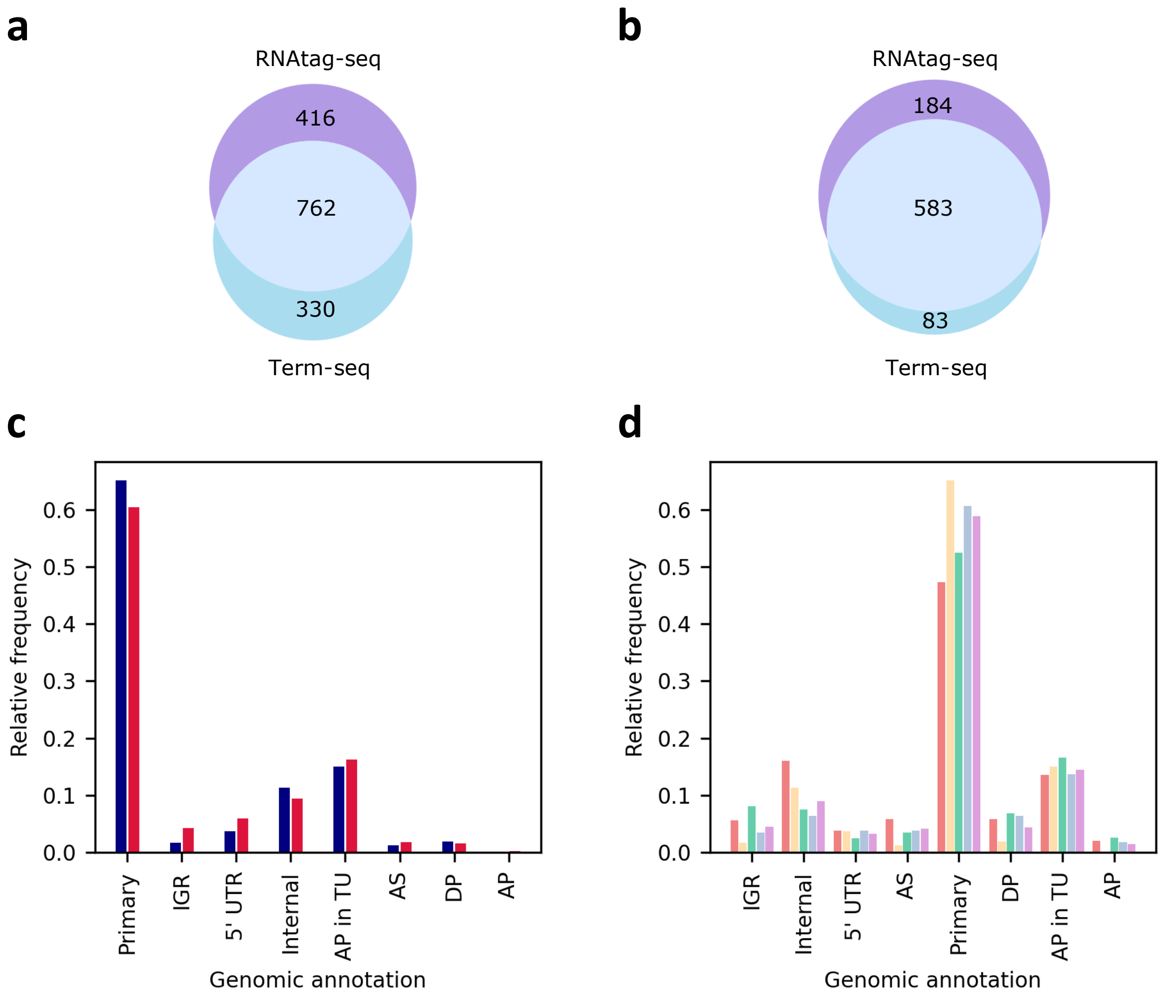

Supplementary Fig. 7. Repertoire of 3’ termini identified by TRS in other bacteria. (a-b) Comparison between the 3’ termini identified by TRS applied to *L.* monocytogenes previously published RNAtag-seq data[7] and term-seq data[8]. Shown is the overlap of the 3’ termini between the two datasets when considering all 3’ termini (a), and when considering only primary 3’ termini (b). (c) Comparison of the 3’ termini genomic annotation assignments of the RNAtag-seq and term-seq datasets in *L. monocytogenes*. Shown by bars is the relative frequency of 3’ termini genomic annotation in the RNAtag-seq dataset (blue) and term-seq dataset (red). (d) Comparison of the genomic annotation of 3’ termini determined by TRS in different bacteria. Shown by bars is the relative frequency of 3’ termini genomic annotation in *E. coli* (red), *L. monocytogenes* (yellow), *S. flexneri* (gree), *S. enterica* (blue), and *K. pneumoniae* (purple). The 3’ termini annotation and abbreviations are according to Fig. 3 in the main text.

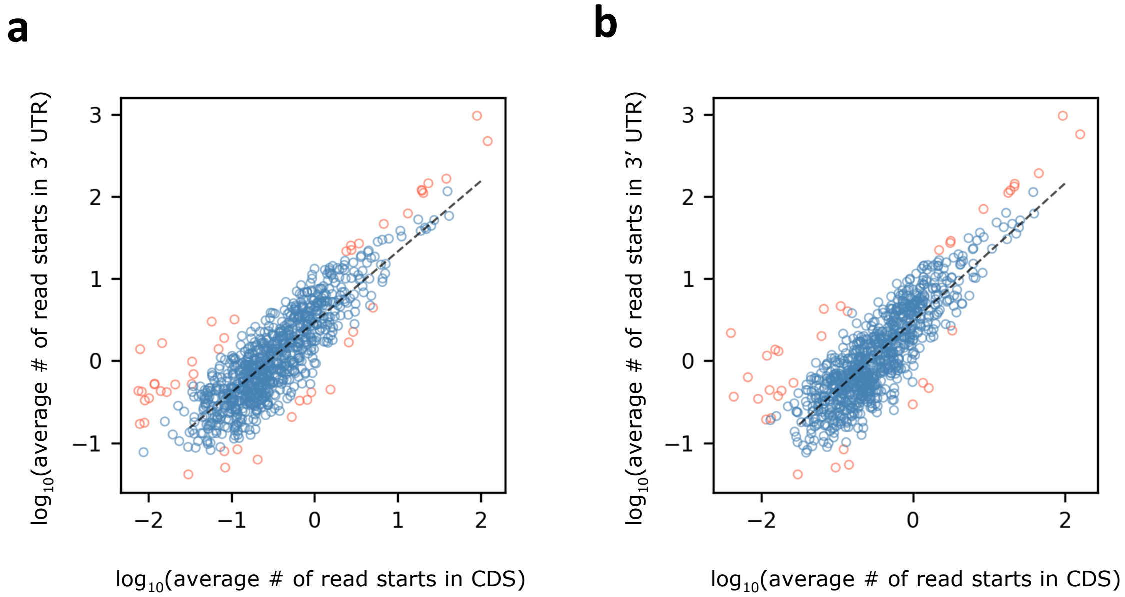

Supplementary Fig. 8. 3' UTR-derived transcripts identified in the *E. coli* K-12 LB RNAtag-seq dataset (related to Fig. 7a in the main text). For each gene with a primary or a distant primary 3' terminus in the LB RNAtag-seq dataset, the log_{10} transformed average number of read starts within the CDS and 3' UTR were computed. Presented are the scatterplots of these values for the two libraries (a and b) not shown in Fig. 7 in the main text, and the regression line fitted (dashed black line). The correlation coefficients are r=0.84 and r=0.85 (p≤ 1.91E-238 and p≤ 2.31E-247, respectively, by two-sided Student's t-test). Genes that were identified as outliers (Methods in main text) are colored red.

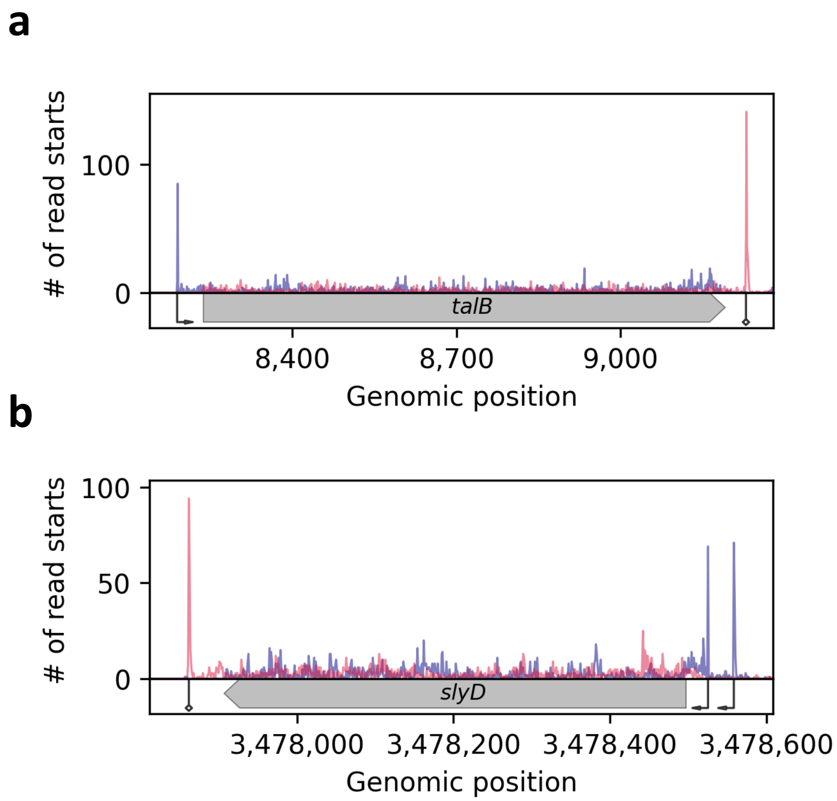

Supplementary Fig. 9. Feasibility of using paired-end read starts to simultaneously determine transcript 5' and 3' boundaries. Presented is the number of read starts of read 1 (red) and read 2 (blue) along the genes *talB* (a) and *slyD* (b) in the Melamed *et al.* 2020[4] dataset (Supplementary Data 1). Transcription start site and termination site identified by SEnd-seq[3] are marked by an arrow and a diamond arrow, respectively.

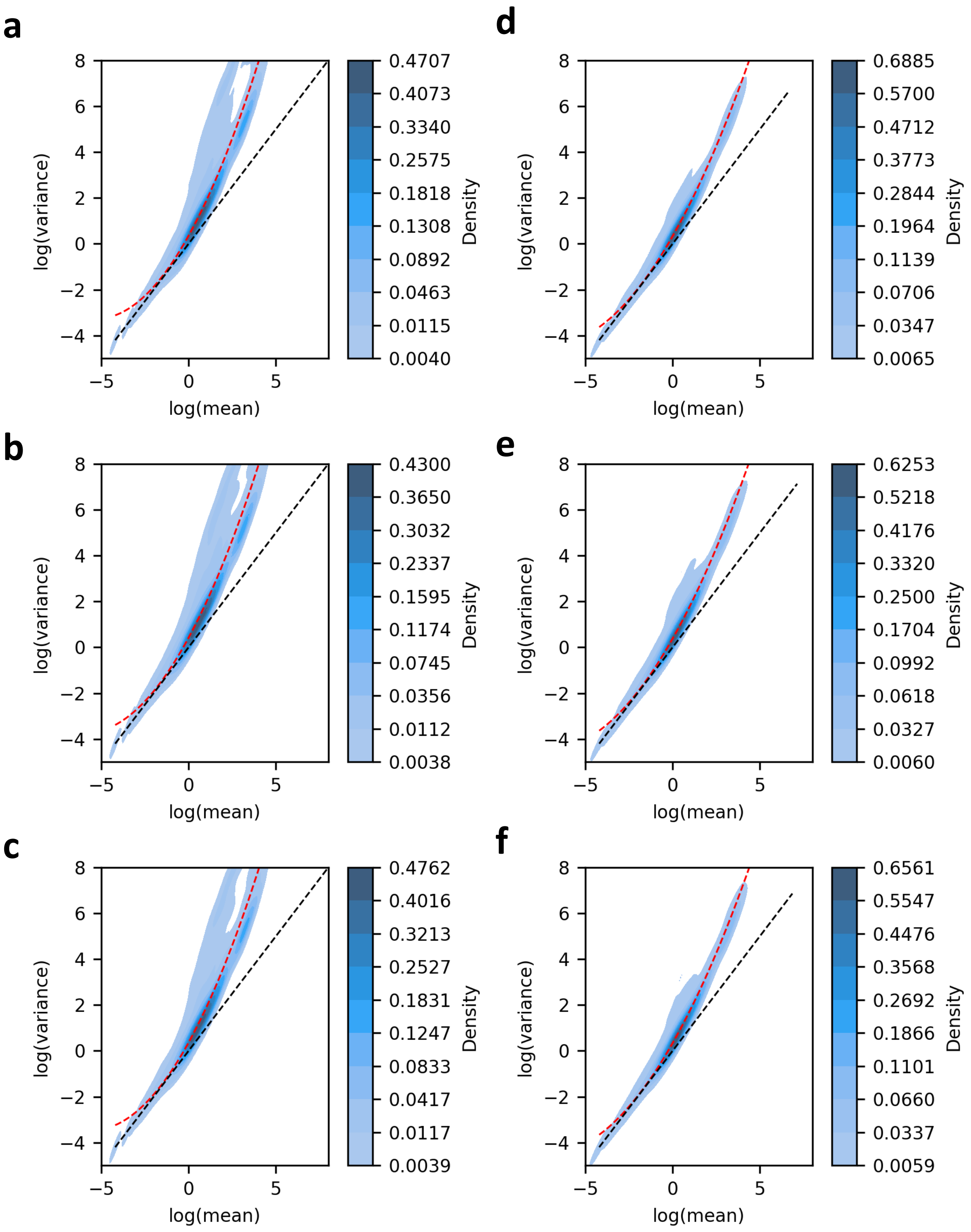

Supplementary Fig. 10. The relationship between the mean and the variance of the number of read starts. Presented is the density map of the mean and variance of number of read starts (natural log transformed) in: (a) Overlapping windows of 70 nucleotides

throughout the genome. (d) Windows of 70 nucleotides downstream $\overline{R_i}$ peaks. For estimation of the variance from the mean, a second-degree polynomial (dashed red line) was fitted and compared to the Poisson model in which the mean equals the variance (dashed black line). (b-c) Same as (a) for the two other replicates. (e-f) Same as (d) for the two other replicates. LB *E. coli* K-12 RNAtag-seq data generated in this study has been used in these analyses.

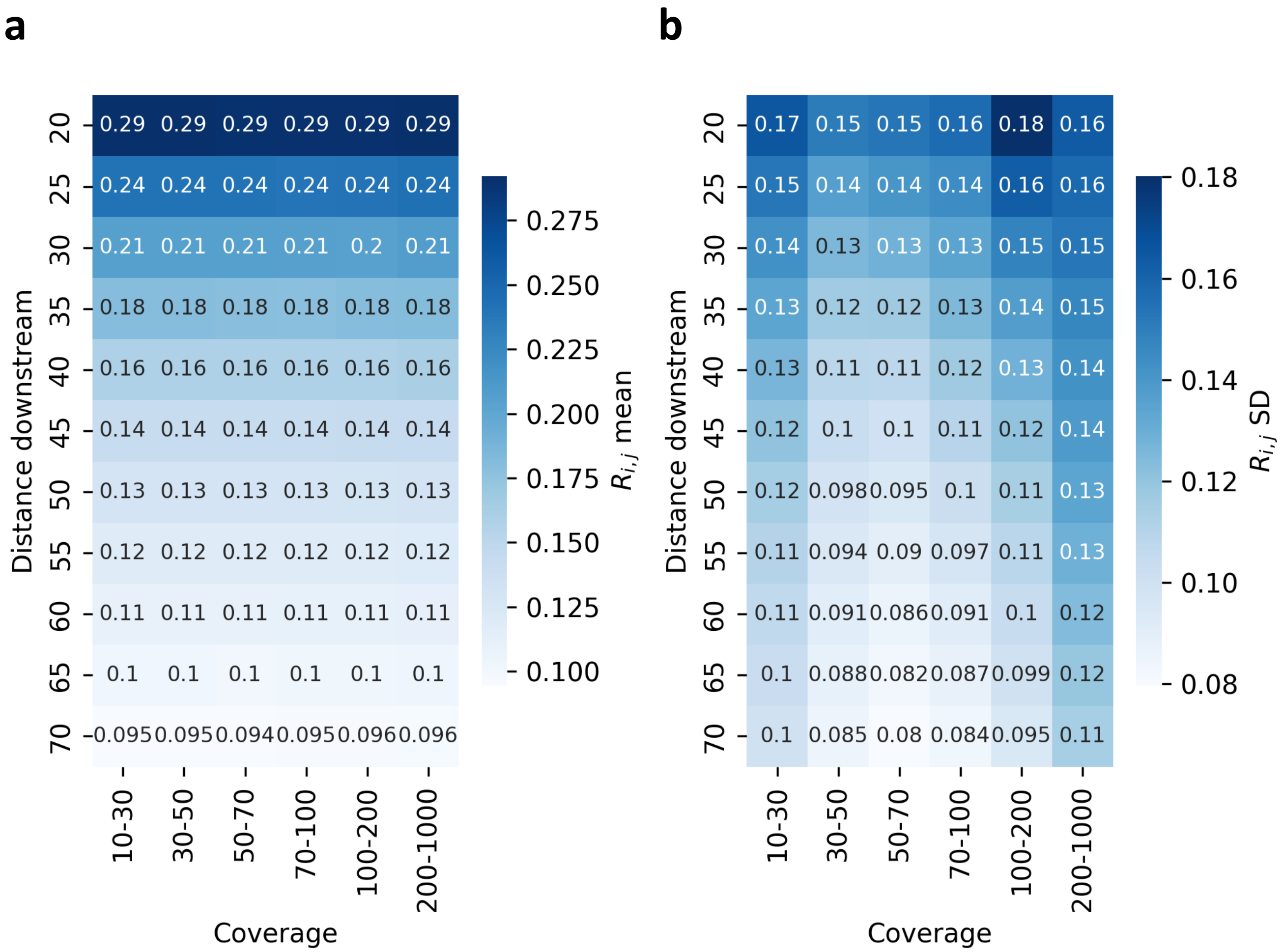

Supplementary Fig. 11. The effect of the read coverage on the value of the statistic $R_{i,j}$. Presented are heatmaps showing the statistic mean (a) and standard deviation (b), as a function of the read coverage in the LB *E. coli* K-12 RNAtag-seq datasets generated in the current study. The identical values along the rows in (a) indicate that the statistic mean is independent of the read start counts (different columns), however it is affected by the choice of the downstream distance (rows). On the other hand, the standard deviation (b) varies for different numbers of read starts.

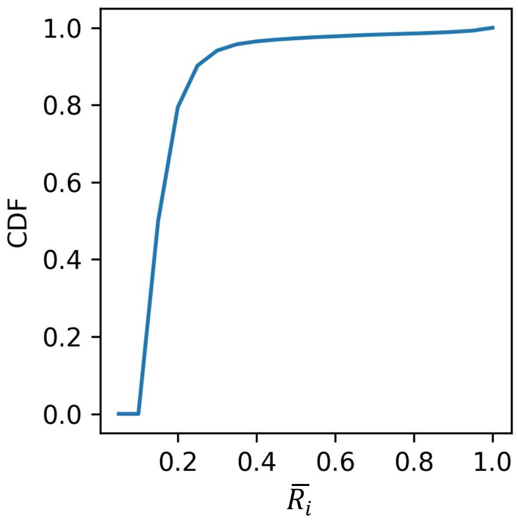

Supplementary Fig. 12. The cumulative distribution function of the mean statistic $\overline{R_i}$. Presented is the cumulative distribution function (CDF) of the mean statistic values across the three libraries of putative 3' termini tested by the algorithm. LB *E. coli* K-12 RNAtag-seq data generated in this study has been used in these analyses.

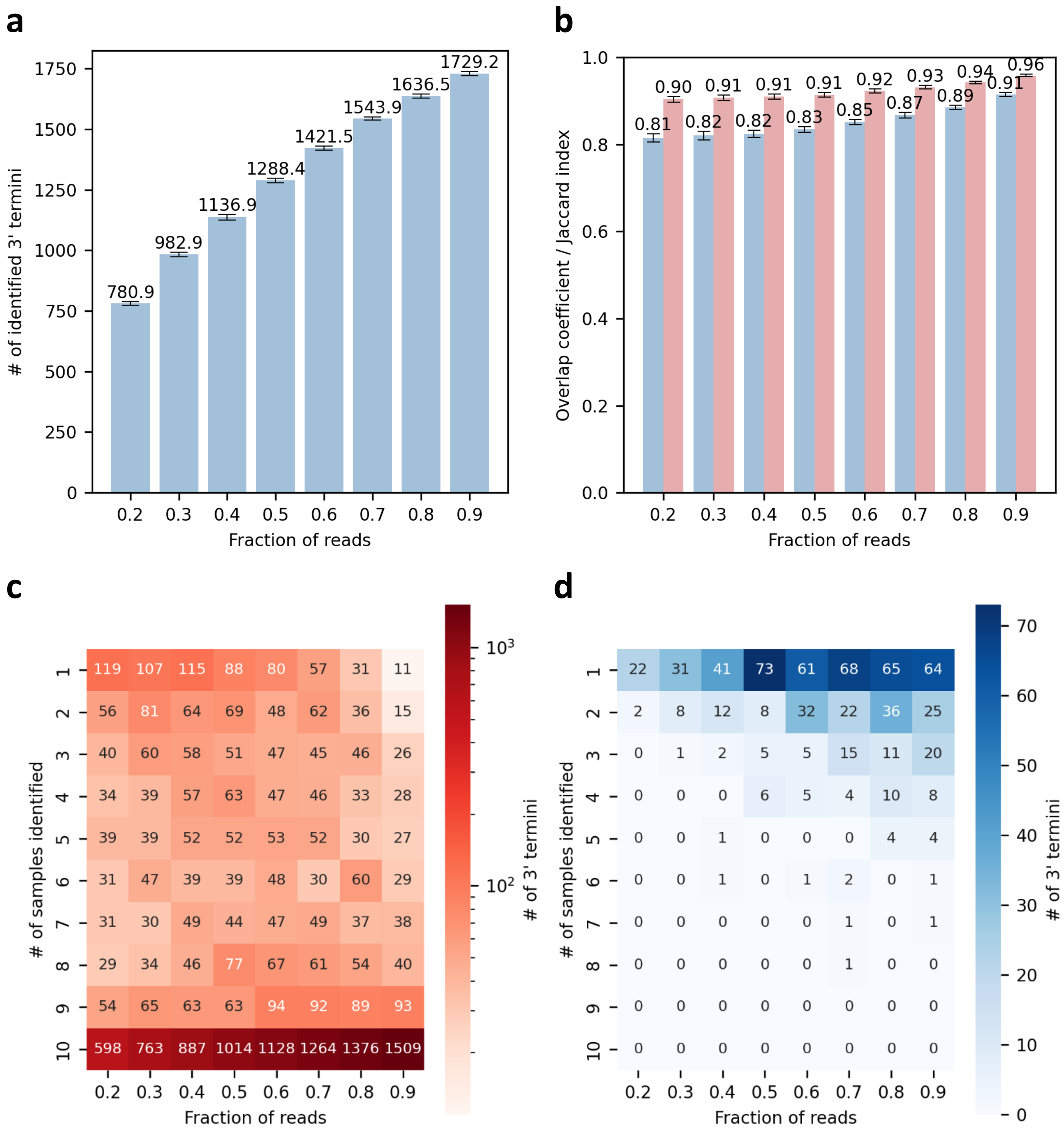

Supplementary Fig. 13. Effect of sequencing depth on the identified 3' termini. To estimate the library size effect on the identified 3' termini, we sampled reads from the three LB *E. coli* K-12 RNAtag-seq replicates at eight different probabilities (0.2-0.9) and applied the algorithm. That is, for each probability we repeated the sampling 10 times,

such that there are 10 different samples of the replicates to which the algorithm was applied. (a) Presented are the mean numbers of 3' termini identified at each depth across the samples. The standard deviation of the samples is presented by error bars. (b) The mean overlap coefficient (red) and Jaccard index (blue) between all pair of samples for each library depth. The standard deviation of the samples is presented by error bars. (c-d) 3' termini across all samples in a specific library depth (columns) were unified, and the number of samples they were identified in was counted (rows). Presented are 3' termini that were found at library full depth (c) and 3' termini that were not found at library full depth (d).

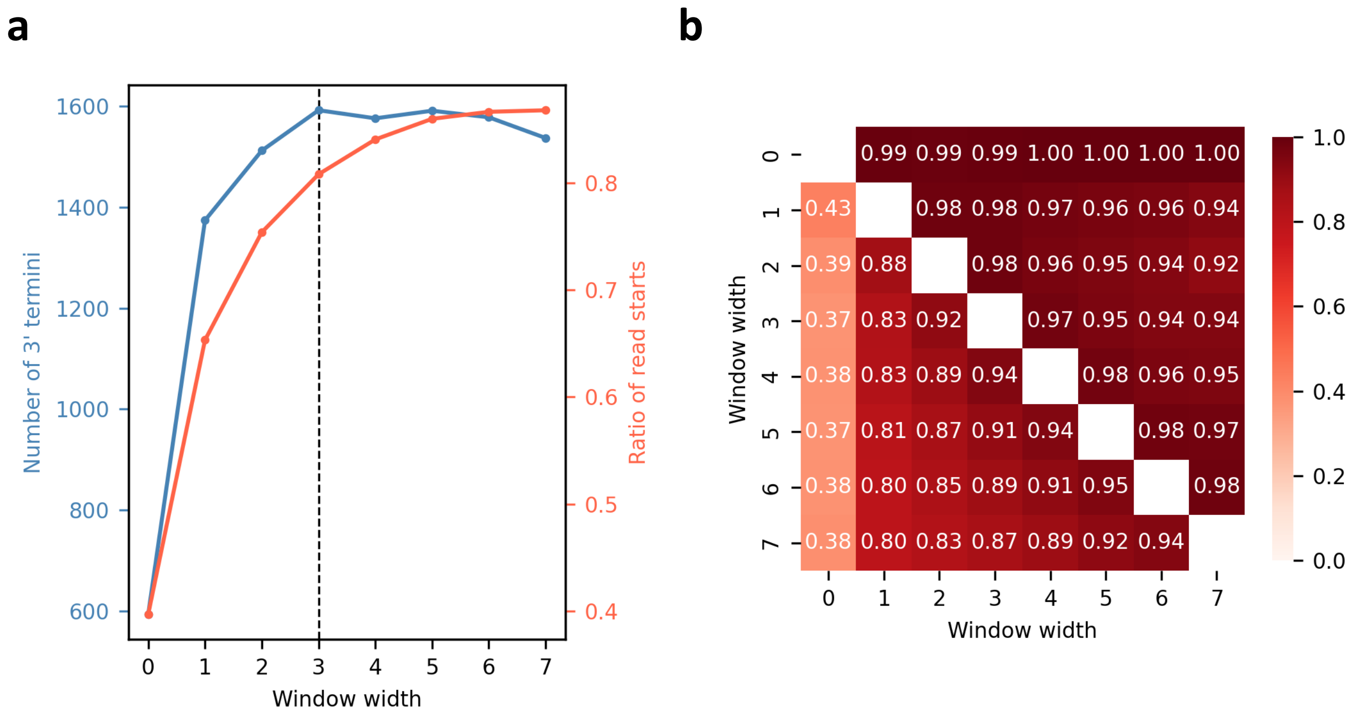

Supplementary Fig. 14. Effect of window size on identified 3' termini.

(a) In TRS the local number of read starts per position is computed using a window of size 2W around every position (where W is the window width). Presented are the numbers of 3' termini identified by the TRS algorithm (blue) for eight values of window width. In parallel, we define a window of size 2W around the prominent position (position within the peak with the maximum number of read starts), and show the ratio between the number of read starts within this window (red) and the number of read starts in an extended window of ±20 nt around the prominent position. The window width used for our final analyses is marked by a black dashed line. (b) Heatmap showing the 3' termini overlap between every two datasets with different window size. The overlap is assessed by two measures: The Jaccard index (lower triangle), which considers the intersection of datasets compared to their union, and the overlap coefficient (upper triangle), which considers the intersection of datasets compared to the smaller dataset size. LB *E. coli* K-12 RNAtag-seq data generated in this study has been used in these analyses.

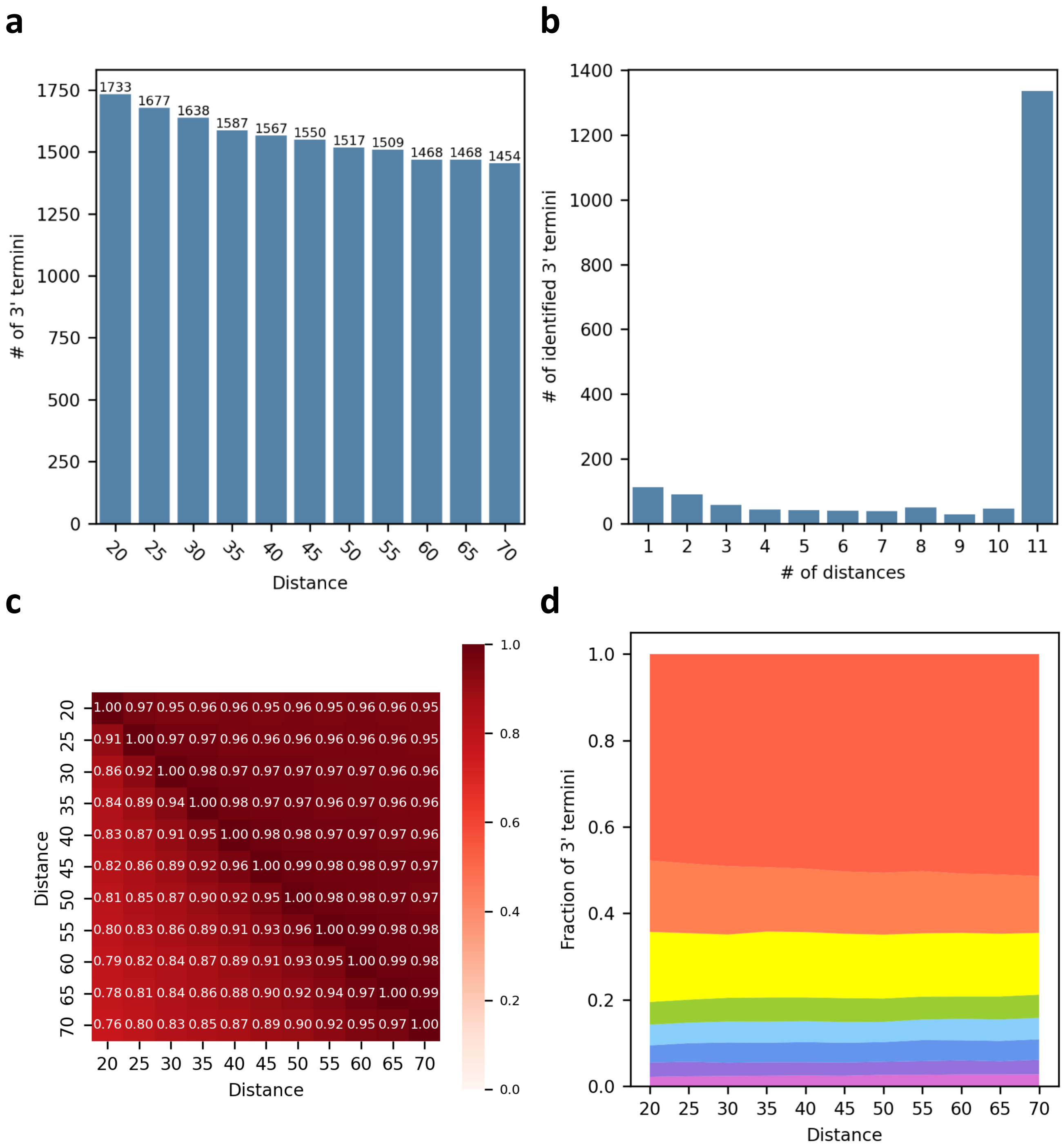

Supplementary Fig. 15. Effect of downstream distance on identified 3' termini. TRS was applied to the LB *E. coli* K-12 RNAtag-seq data with 11 values of downstream distance (denoted by D in the algorithm) to measure the effect of downstream distance on the number and identity of determined 3' termini. (a) Presented is the number of 3' termini identified for different values of the downstream distance. (b) Presented are the

numbers of downstream distances in which each 3’ terminus was identified (number of distances). (c) Heatmap showing the 3’ termini overlap between every two downstream distances by two measures: The Jaccard index (lower triangle), which considers the intersection of datasets compared to their union, and the overlap coefficient (upper triangle), which considers the intersection of datasets compared to the smaller dataset size. (d) The genomic annotation distribution of 3’ termini identified for different values of the downstream distance. The colors correspond to the following annotations from top to bottom: Primary, internal, AP in TU, IGR, DP, AS, 5’ UTR and AP. For the annotations and abbreviations see Fig. 3 in the main text.

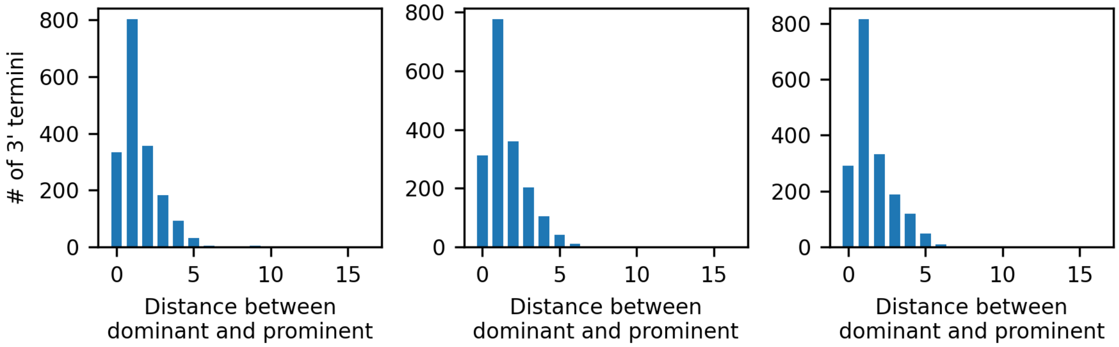

Supplementary Fig. 16. Distribution of distances between dominant and prominent positions of statistically significant $\overline{R_\iota}$ peaks. For each of the LB *E. coli* K-12 RNAtag-seq libraries, shown are the distributions of absolute distances between the position where $\overline{R_\iota}$ was maximal (dominant position) and the position with highest read start count (prominent position). Included are positions of statistically significant $\overline{R_\iota}$ peaks.

Supplementary References

1. Dar, D. & Sorek, R. High-resolution RNA 3'-ends mapping of bacterial Rho-dependent transcripts. *Nucleic Acids Res* **46**, 6797-6805 (2018).
2. Adams, P.P. et al. Regulatory roles of Escherichia coli 5' UTR and ORF-internal RNAs detected by 3' end mapping. *eLife* **10** (2021).
3. Ju, X., Li, D. & Liu, S. Full-length RNA profiling reveals pervasive bidirectional transcription terminators in bacteria. *Nature microbiology* **4**, 1907-1918 (2019).
4. Melamed, S., Adams, P.P., Zhang, A., Zhang, H. & Storz, G. RNA-RNA Interactomes of ProQ and Hfq Reveal Overlapping and Competing Roles. *Mol Cell* **77**, 411-425 (2020).
5. Goldberger, O., Livny, J., Bhattacharyya, R. & Amster-Choder, O. Wisdom of the crowds: A suggested polygenic plan for small-RNA-mediated regulation in bacteria. *iScience* **24**, 103096 (2021).
6. Kavita, K. et al. Multiple in vivo roles for the C-terminal domain of the RNA chaperone Hfq. *Nucleic Acids Res* **50**, 1718-1733 (2022).
7. Avican, K. et al. RNA atlas of human bacterial pathogens uncovers stress dynamics linked to infection. *Nature communications* **12**, 3282 (2021).
8. Dar, D. et al. Term-seq reveals abundant ribo-regulation of antibiotics resistance in bacteria. *Science* **352**, aad9822 (2016).
9. Huerta-Cepas, J. et al. eggNOG 5.0: a hierarchical, functionally and phylogenetically annotated orthology resource based on 5090 organisms and 2502 viruses. *Nucleic Acids Res* **47**, D309-D314 (2019).
10. Clarke, J.E., Kime, L., Romero A., D. & McDowall, K.J. Direct entry by RNase E is a major pathway for the degradation and processing of RNA in *Escherichia coli*. *Nucleic Acids Res* **42**, 11733-11751 (2014).
11. Altuvia, Y. et al. In vivo cleavage rules and target repertoire of RNase III in Escherichia coli. *Nucleic Acids Res* **46**, 10530-10531 (2018).
12. Fu, Y., Wu, P.H., Beane, T., Zamore, P.D. & Weng, Z. Elimination of PCR duplicates in RNA-seq and small RNA-seq using unique molecular identifiers. *BMC Genomics* **19**, 531 (2018).
13. Rochette, N.C. et al. On the causes, consequences, and avoidance of PCR duplicates: Towards a theory of library complexity. *Mol Ecol Resour* (2023).
14. Danecek, P. et al. Twelve years of SAMtools and BCFtools. *GigaScience* **10** (2021).
15. Bar, A., Argaman, L., Altuvia, Y. & Margalit, H. Prediction of Novel Bacterial Small RNAs From RIL-Seq RNA-RNA Interaction Data. *Frontiers in microbiology* **12**, 635070 (2021).
16. Lorenz, R. et al. ViennaRNA Package 2.0. *Algorithms Mol Biol* **6**, 26 (2011).
