# Supplementary material for: TRS: a method for determining transcript termini from RNAtag-seq sequencing data
Source: Nat Commun. 2023 Nov 29;14:7843. doi: 10.1038/s41467-023-43534-2 (PMC10687069; doi:10.1038/s41467-023-43534-2)
Supplement: Supplementary file 3 — Description of Additional Supplementary Files [file 41467_2023_43534_MOESM3_ESM.pdf]

Description of Additional Supplementary Files

File Name: Supplementary Data 1
Description: Previously published sequencing datasets that were analyzed by TRS (Excel file).
This file describes the previously published sequencing libraries analyzed in this study. The file lists for each analysis which datasets were used (accessions), the bacterial strain, the reference genome and genome annotation used for the analysis, the sequencing methodology and the corresponding sheet in Supplementary Data 2.

File Name: Supplementary Data 2
Description: 3’ termini determined by TRS for datasets analyzed in this study (Excel file).
The file lists the 3’ termini identified by applying TRS to the various previously published sequencing datasets described in Supplementary Data 1.

File Name: Supplementary Data 3
Description: 3’ termini identified by applying TRS to RNAtag-seq and term-seq data of the same RNA sample (Excel file).
The file lists 3’ termini identified by applying TRS to RNAtag-seq and term-seq data of RNA from *E. coli* grown to exponential phase in rich (LB) and minimal (EG) media.

File Name: Supplementary Data 4
Description: 3’ UTR – CDS expression analysis (Excel file).
The file summarizes the analysis associating the expression level in the CDS of transcripts with the expression level in the 3’ UTR determined by the 3’ termini identified in this study.

File Name: Supplementary Data 5
Description: EPEC condition-dependent changes in 3’ termini (Excel file).
The file lists the top 50% premature 3’ termini that presented the highest difference in the mean statistic $\overline{R_\iota}$ between EPEC grown in LB to stationary phase and EPEC grown in DMEM to exponential phase.
